# Protection by simvastatin on hyperglycemia-induced endothelial dysfunction through inhibiting NLRP3 inflammasomes

**DOI:** 10.18632/oncotarget.20443

**Published:** 2017-08-24

**Authors:** Zhen-Huan Lv, Trinh Anh Phuong, Shi-Jie Jin, Xiao-Xue Li, Ming Xu

**Affiliations:** ^1^ Department of Clinical Pharmacy, School of Basic Medical Sciences and Clinical Pharmacy, China Pharmaceutical University, Nanjing 210009, China; ^2^ School of Pharmacy, Zhejiang Chinese Medical University, Hangzhou 311400, China; ^3^ Department of Pathology, Medical School of Southeast University, Nanjing 210009, China

**Keywords:** simvastatin, diabetes, NLRP3 inflammasome, HMGB1, vascular endothelial permeability

## Abstract

Recent studies have demonstrated that NLRP3 inflammasome complex acts as pivotal elements to initiate inflammatory responses and plays an important role in the dysfunction of cardiovascular complications. Meanwhile, simvastatin prevents vascular endothelial dysfunction from inflammasome invasion contributing to reduce cardiovascular risk. However, Whether or not the simvastatin improves vascular endothelial barrier function through inhibiting the activation of NLRP3 inflammasome pathway remains unknown. Here, we explored the role and mechanisms of simvastatin in the activation of NLRP3 inflammasome which are involved in vascular endothelial hyperpermeability causing by the disruption of tight junction protein ZO-1 and adherens junction protein VE-Cadherin, an early initiation of cardiovascular complication. Our results found that high glucose significantly induced the formation and activation of NLRP3 inflammasome through NADPH oxidase-dependent reactive oxygen species (ROS) formation, associated with vascular endothelial hyperpermeability causing by ZO-1 and VE-Cadherin disruption in the rat aortic endothelial cells (RAECs). Simvastatin treatment remarkably abolished vascular endothelial hyperpermeability and enhanced the protein expression of ZO-1 and VE-Cadherin through NLRP3 inflammasome. Mechanistically, the inhibitory role of simvastatin endothelial hyperpermeability is attributed to the decreased release of cytoplasmic high mobility group box protein-1 (HMGB1) derived from endothelial NLRP3 inflammasome activation. We further confirm the protective role of simvastatin on vascular leakage in the heart of diabetic rats injected with Evans blue dye, which was associated with HMGB1 release in the serum. Collectively, the mechanism of simvastatin treatment alleviating vascular endothelial permeability dysfunction may be through inhibiting the NLRP3 inflammasome-dependent HMGB1 release in RAECs.

## INTRODUCTION

Recently studies have demonstrated a central role of inflammasome in the pathogenesis of cardiovascular diseases including atherosclerosis [1], hypertension [2], vascular inflammation [3] and cardiac remodeling [4]. In particular, the NLRP3 inflammasome as an important sensor is involved in the vascular endothelial pathological process, which assembly activate caspase-1 leading to the processing of bio-active IL-1β secretion in diabetes [5, 6]. On the other hand, accumulating evidence indicate that inflammasome activation can cause cell dysfunction or injury via non-canonical actions through pyroptosis, interference with cytoskeleton arrangement, lipid handling, or direct regulation of synthesis, metabolism, or secretion of functional proteins [7, 8]. In this respective, we recently reported that such non-canonical role of inflammasome was involved in endothelial dysfunction and injury during obesity [9, 10]. Therefore, there are great potential clinical implications in understanding the mechanisms underlying inflammasome regulation of vascular endothelial function by pharmacological interventions.

Statins, inhibitors of 3-hydroxy-3-methylglutaryl coenzyme A (HMG-CoA) reductase, are extensively used to lower plasma cholesterol to treat atherosclerosis [11]. However, recent observations demonstrate that statins have anti-inflammation and immunomodulatory properties, improve nitric oxide bioavailability and prevent endothelial dysfunction that are well beyond their lipid-lowering properties [12, 13]. Our previous results indicate that simvastatin may stabilize vascular smooth muscle cells in a more contractile phenotype and thereby prevent these cells from proliferation and growth [14]. On the contrary, epidemiological evidences have revealed that several statins were associated with small, but significant increased risk of new onset diabetes [15, 16]. Despite the emergence of new diabetes, it is clear that the risk–benefit ratio for cardiovascular disease events is strongly in favor of statin therapy in those diseases at risk. Here, we detected the protective role of statins on diabetes-induced vascular injury and the underlying mechanisms. It remains unknown whether simvastatin, a clinically widely used statin, could improve vascular endothelial injury via NLRP3 inflammasome. The present study hypothesized that inhibited NLRP3 inflammasome by simvastatin is an important mechanism leading to their preventive effects on vascular endothelial cells.

To test this hypothesis, we treated vascular endothelial cells and rats with simvastatin in diabetic condition and observed the NLRP3 inflammasome and changes of vascular permeability which is a common pathogenic change during the development of chronic inflammation such as type 2 diabetes [17]. Our results indicate that simvastatin inhibited the NLRP3 inflammasome pathway and thereby decreased the secretion of HMGB1 from vascular endothelial cells. We also demonstrated that this decrease of HMGB1 secretion results in the upregulation of tight junction protein ZO-1 and adherens junction VE-Cadherin at cell junctions associated with recovery of vascular hyperpermeability by simvastatin. These studies thus revealed a potential mechanism for the beneficial action of statins in diabetic vascular complication.

## RESULTS

### High glucose induces NLRP3 inflammasome formation and activation in RAECs

**Figure 1:**
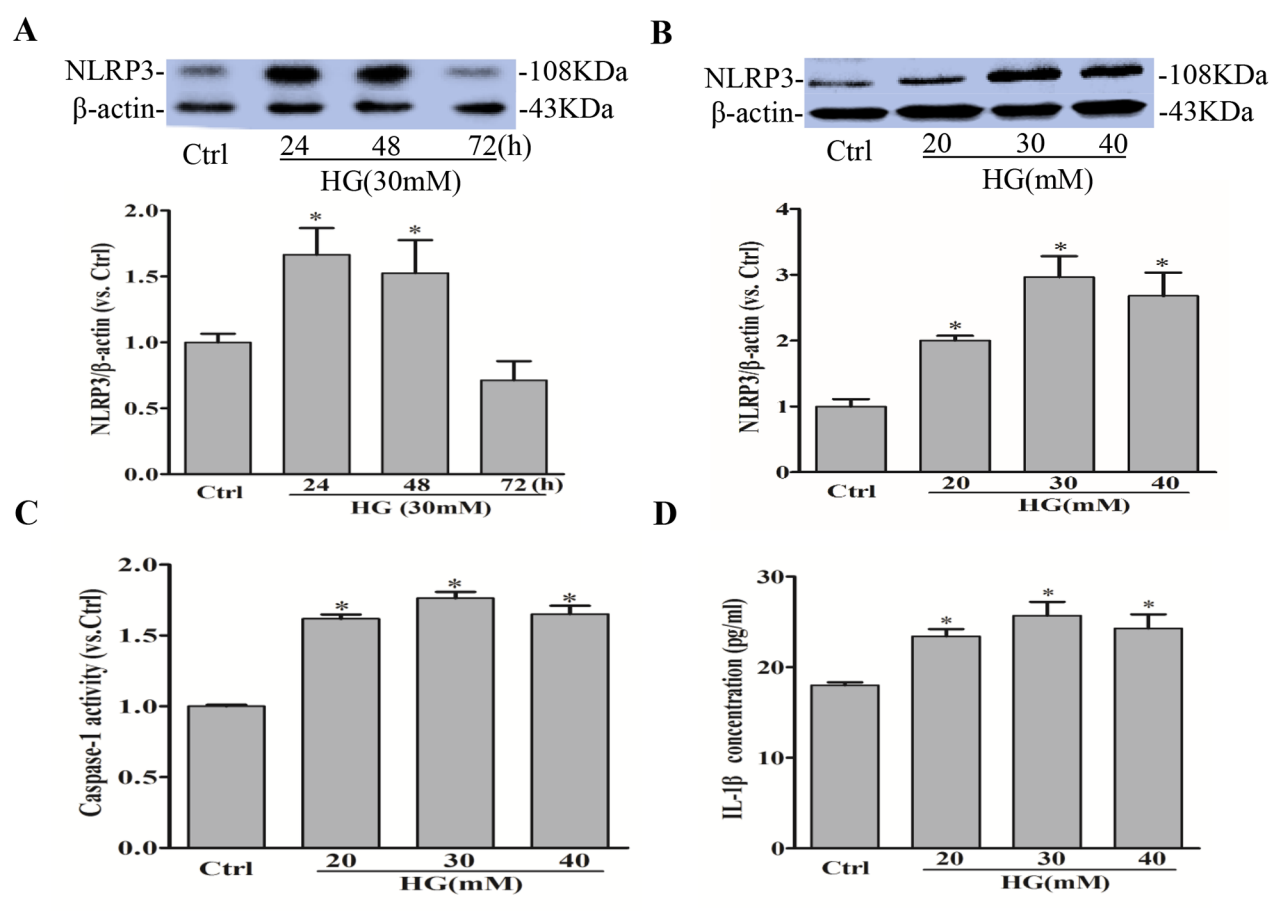
High glucose induces NLRP3 inflammasome activation in RAECs Representative Western blot gel documents and summarized data showing the protein expression of NLRP3 in RAECs for 24, 48, 72h of exposure to high glucose **(A)**, and 20, 30, 40 mM glucose **(B)**. **(C)** Summarized data showing caspase-1 activity in RAECs. **(D)** Summarized data showing IL-1β production in RAECs. *P<0.05 *vs.* Control (Ctrl) (n=3).

NLRP3 inflammasome serves as a principal machinery to trigger the inflammatory responses in a variety of mammalian cells including endothelial cells [18]. We first confirm that high glucose induced NLRP3 inflammasome formation and activation in RAECs. As shown in Figure 1A-1B, high glucose time-dependently and dose-dependently increased the expression of NLRP3 protein in RAECs, which was highest under 30mmol/l glucose at 24h. We further found that glucose dose-dependently enhanced caspase-1 activity in RAECs exposed under 20, 30, and 40 mmol/l glucose (Figure 1C), which is consist with the increase of IL-1β release, a primary caspase-1 substrate, in cell supernatants as measured by commercially available ELISA kit (Figure 1D).

### Effect of simvastatin on high glucose-induced NLRP3 expression and oligomerization

**Figure 2:**
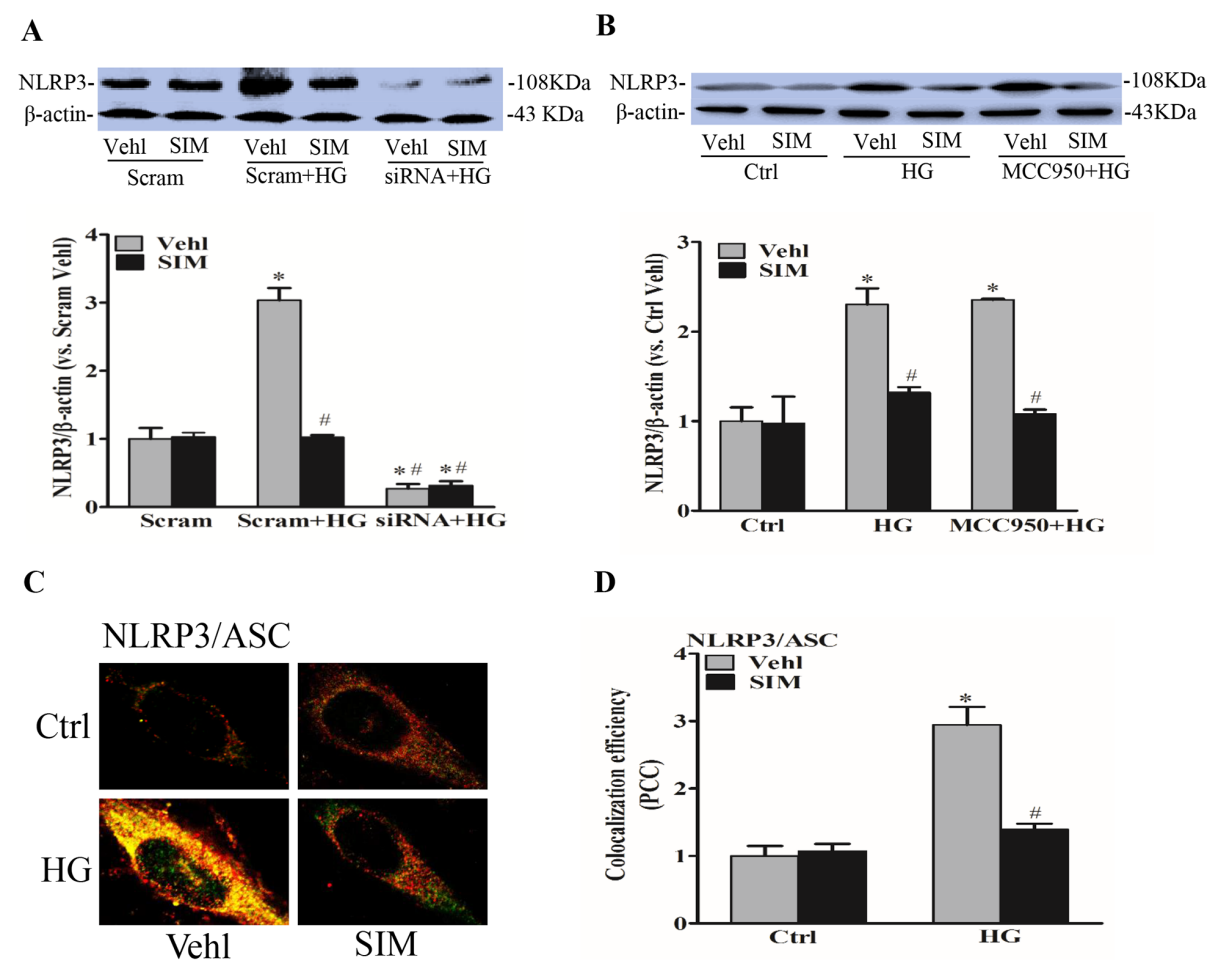
Simvastatin inhibited high glucose-induced NLRP3 expression and oligomerization RAECs were incubated with high glucose for 24h, which was treated with simvastatin (SIM, 5μM) in the presence or absence of the transfection of NLRP3 siRNA or pretreatment of MCC950 (15nM). **(A, B)** Representative Western blot gel documents and summarized data showing the protein expression of NLRP3. Representative confocal fluorescence images **(C)** and the colocalization efficiency **(D)** showing the colocalization of NLRP3 with ASC. *P<0.05 *vs.* Scram Vehicle (Vehl) or Ctrl Vehl; ^#^*P*<0.05 *vs.* HG treated group (n=4).

Next, we observed the role of simvastatin on NLRP3 inflammasome activation in RAECs. RAECs were treated with different doses simvastatin, and it is found that 5μM simvastatin had the most significant effects on NLRP3 protein expression (not shown) with no significant effects on cell viability with MTT assay (Supplementary Figure 1). In addition, NLRP3 siRNA was transfected into RAECs to silence NLRP3 gene, resulting in 70% inhibition of NLRP3 protein expression (Figure 2A). MCC950 as an inhibitor of NLRP3 inflammasome had no inhibitory effects on the expression of NLRP3 protein (Figure 2B). Simultaneously, we analyzed the colocalization of inflammasome components by confocal microscopy. As shown in Figure 2C and 2D, the colocalization of NLRP3 with ASC was markedly increased in response to high glucose priming for 24 hours, indicating the aggregation or assembly of these inflammasome molecules. Under simvastatin treatment, the colocalization level of NLRP3 and ASC was significantly decreased, which further certified that simvastatin had an inhibitory effects on NLRP3 activation. In addition, we also found that the activation of NLRP3 inflammasome induced by high glucose shared similar characteristics with LPS and ATP (Supplementary Figure 1).

### Effect of simvastatin on high glucose-induced caspase-1 activity and IL-1β release

**Figure 3:**
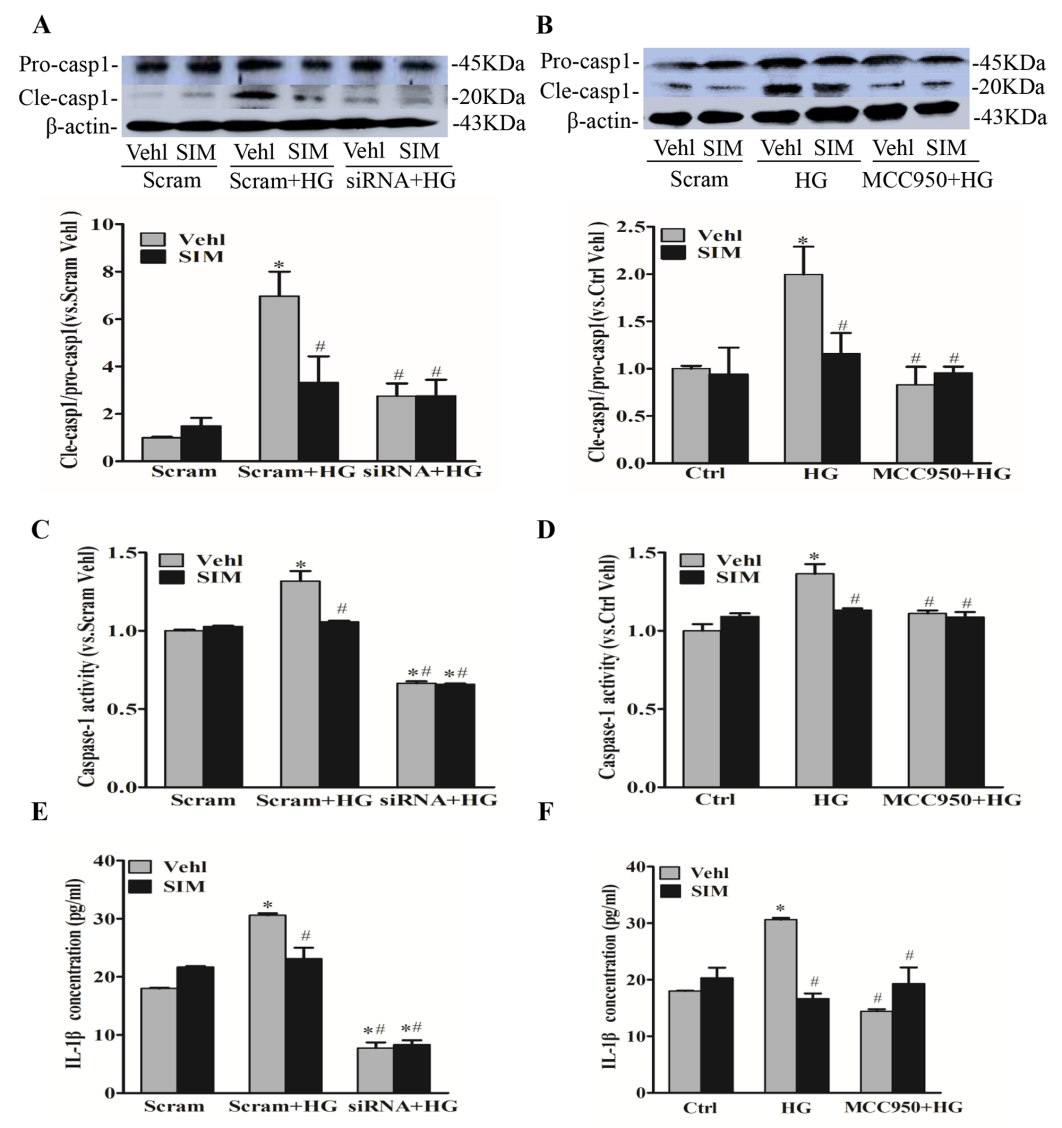
Simvastatin inhibited high glucose-induced caspase-1 activity and IL-1β release **(A, B)** Representative Western blot gel documents and summarized data showing the protein expression of pro-caspase-1 (Pro-casp1) and cleaved caspase-1 (Cle-casp1). **(C, D)** Summarized data showing caspase-1 activity in RAECs. **(E, F)** Summarized data showing IL-1β production in RAECs. *P<0.05 *vs.* Scram Vehicle (Vehl) or Ctrl Vehl; ^#^*P*<0.05 *vs.* HG treated group (n=4).

When the Nlrp3 inflammasome complex is formed, caspase-1 is activated to cleave its substrates including the precursors of inflammatory cytokine interleukin IL-1β. Thus,we also tested caspase-1 activity and IL-1β production. After NLRP3 siRNA transfection or MCC950 pretreatment, RAECs were subsequently stimulated with high glucose (30mM), and after 6 hours, cells were incubated with simvastatin for 18 hours. We found that the expression of cleaved caspase-1(Figure 3A and 3B), caspase-1 activity (Figure 3C and 3D) and the release of IL-1β (Figure 3E and 3F) were dramatically suppressed by the pretreatment with simvastatin as well as NLRP3 siRNA or MCC950, separately. However, simvastatin combined with MCC950 or NLRP3 siRNA showed no additive effects on caspase-1 activity and the release of IL-1β, which confirmed that simvastatin indeed inhibited the activation of NLRP3 inflammasome induced by high glucose.

### Effect of simvastatin on NADPH oxidase-dependent ROS formation

**Figure 4:**
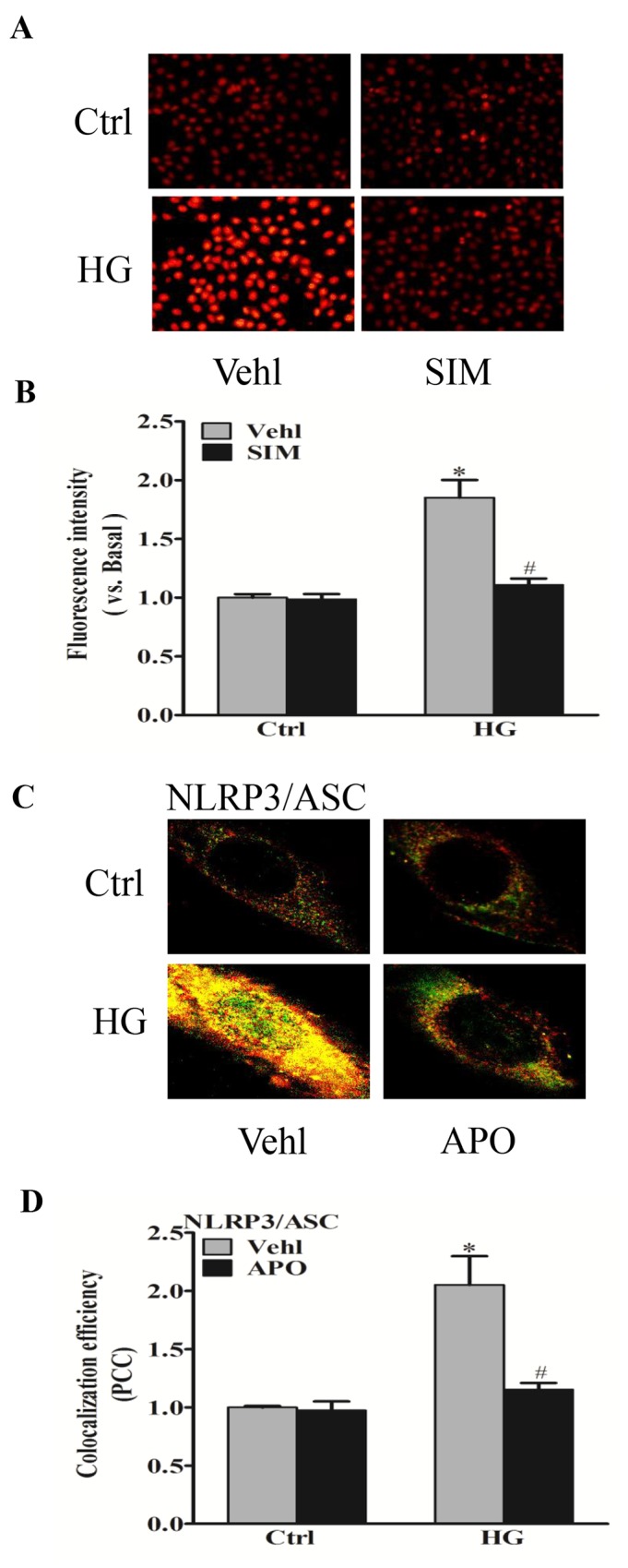
Simvastatin inhibited high glucose-induced NADPH oxidase-dependent O_2_^−^ formation RAECs were stained by DHE, and typical representative fluorescent images for DHE staining **(A)** and summarized data showing the role of simvastatin (SIM, 5μM) on O_2_^−^ production in RAECs incubated with HG **(B)**. Representative confocal fluorescence images **(C)** and the colocalization efficiency **(D)** showing the role of NADPH oxidase inhibitor, apocynin (APO, 10^-5^M) on the colocalization of NLRP3 with ASC. *P<0.05 *vs.* Ctrl Vehl; ^#^*P*<0.05 *vs.* HG treated group (n=4).

It was reported that endogenous superoxide (O_2_^−^) produced primarily contributes to NLRP3 inflammasome formation and activation [19]. Thus, we detected the effects of simvastatin on NADPH oxidase-dependent O_2_^−^ formation. O_2_^−^ oxidizes DHE to produce a strong red fluorescence, thus DHE is widely used for intracellular O_2_^−^ production. As showed in Figure 3A, simvastatin significantly blocked the increase in intracellular red fluorescence induced by high glucose compared with control. The inhibitory effects of NADPH oxidase inhibitor, apocynin (APO) on the oligomerization of NLRP3 and ASC induced by high glucose were also observed (Figure 4B), suggesting that simvastatin inhibited the NLRP3 inflammasome activation via NADPH oxidase-dependent ROS production in RAECs.

### Effect of simvastatin on high glucose-induced tight and adherens junction proteins

**Figure 5:**
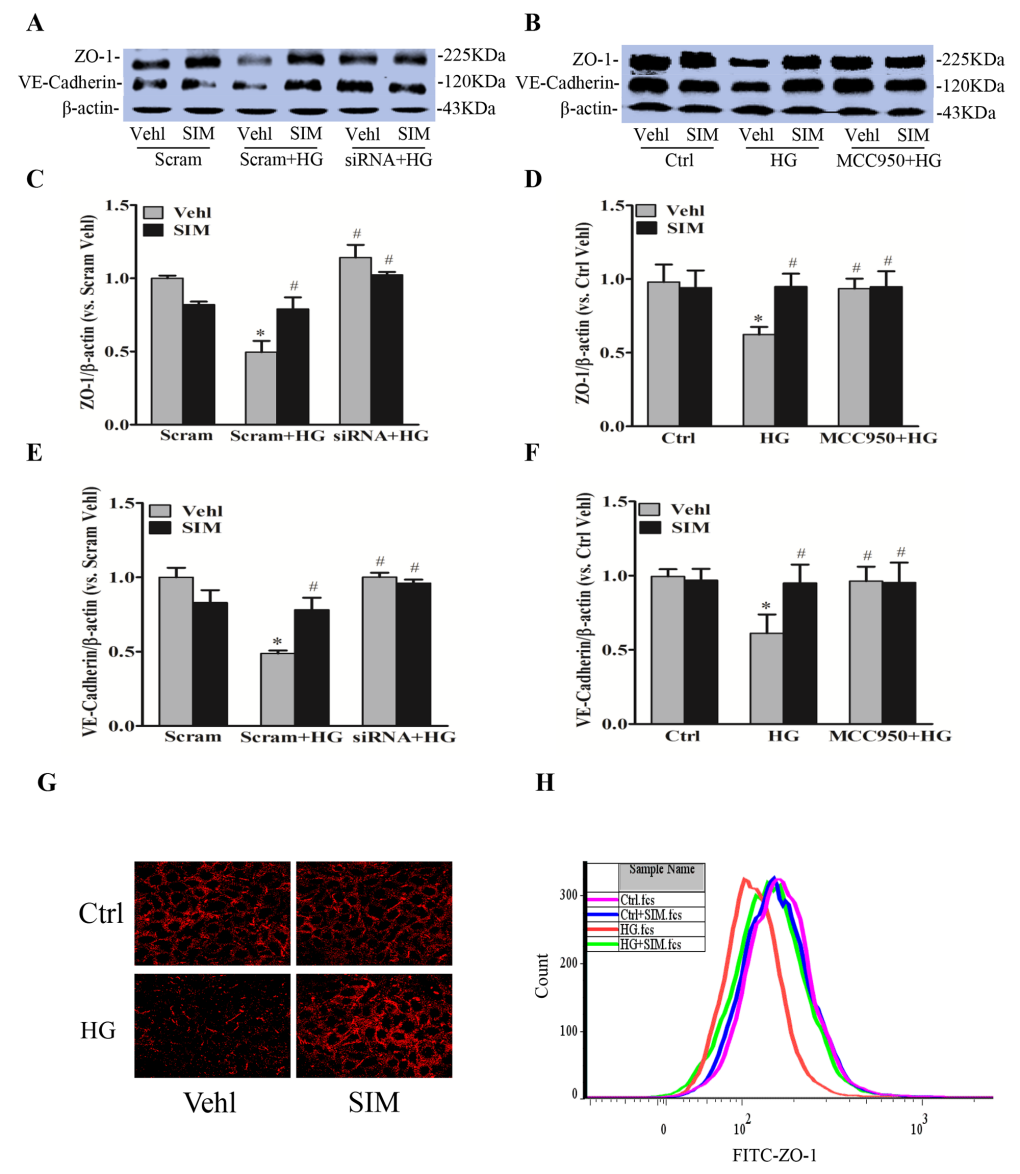
Simvastatin reversed protein expression of high glucose-induced ZO-1 and VE-Cadherin RAECs were incubated with high glucose for 24h, which was treated with simvastatin (SIM, 5μM) in the presence or absence of NLRP3 siRNA or MCC950 (15nM). Representative Western blot gel documents **(A, B)** and summarized data showing the protein expression of ZO-1 **(C, D)** and VE-Cadherin **(E, F)**. **(G)** Representative fluorescence images showing the cell membrane fluorescence of ZO-1 from at least three independent experiments. **(H)** Frequency histogram of ZO-1 in the membranes showing the protein expression of ZO-1 by flowcytometry. *P<0.05 *vs.* Scram Vehl or Ctrl Vehl; ^#^*P*<0.05 *vs.* HG treated group (n=4).

Endothelium as a permeability barrier is composed of endothelial cells, which connected by junction proteins. Tight junction protein ZO-1 and adherens junction protein VE-Cadherin play an important role in regulating paracellular permeability of inter-endothelial junctions [8]. We further examined whether NLRP3 inflammasome was responsible for the role of simvastatin on disassembly of junction proteins caused by high glucose in RAECs. As shown in Figure 5A and 5D, high glucose treatment markedly degraded the protein expression of tight junction ZO-1 and adherens junction VE-Cadherin at cell junctions in endothelial cell monolayers, which were reversed by simvastatin. The activation of NLRP3 inflammasome caused disassembly of junction proteins as showed by the recovery of protein expression of ZO-1 (Figure 5B and 5E) and VE-Cadherin (Figure 5C and 5F) by MCC950 or NLRP3 siRNA compared with high glucose group. However, MCC950 or NLRP3 siRNA showed no further effects on the protein level of ZO-1 and VE-Cadherin followed by the pretreatment with simvastatin, which suggested that NLRP3 inflammasome mediated the inhibition of simvastatin on high glucose-induced tight and adherens junction proteins disruption. Such amelioration of simvastatin in tight and adherens junction proteins was further confirmed by immunocytochemistry (Figure 5G) and flow cytometry assay (Figure 5H).

### Effects of simvastatin on high glucose-induced endothelial hyperpermeability

**Figure 6:**
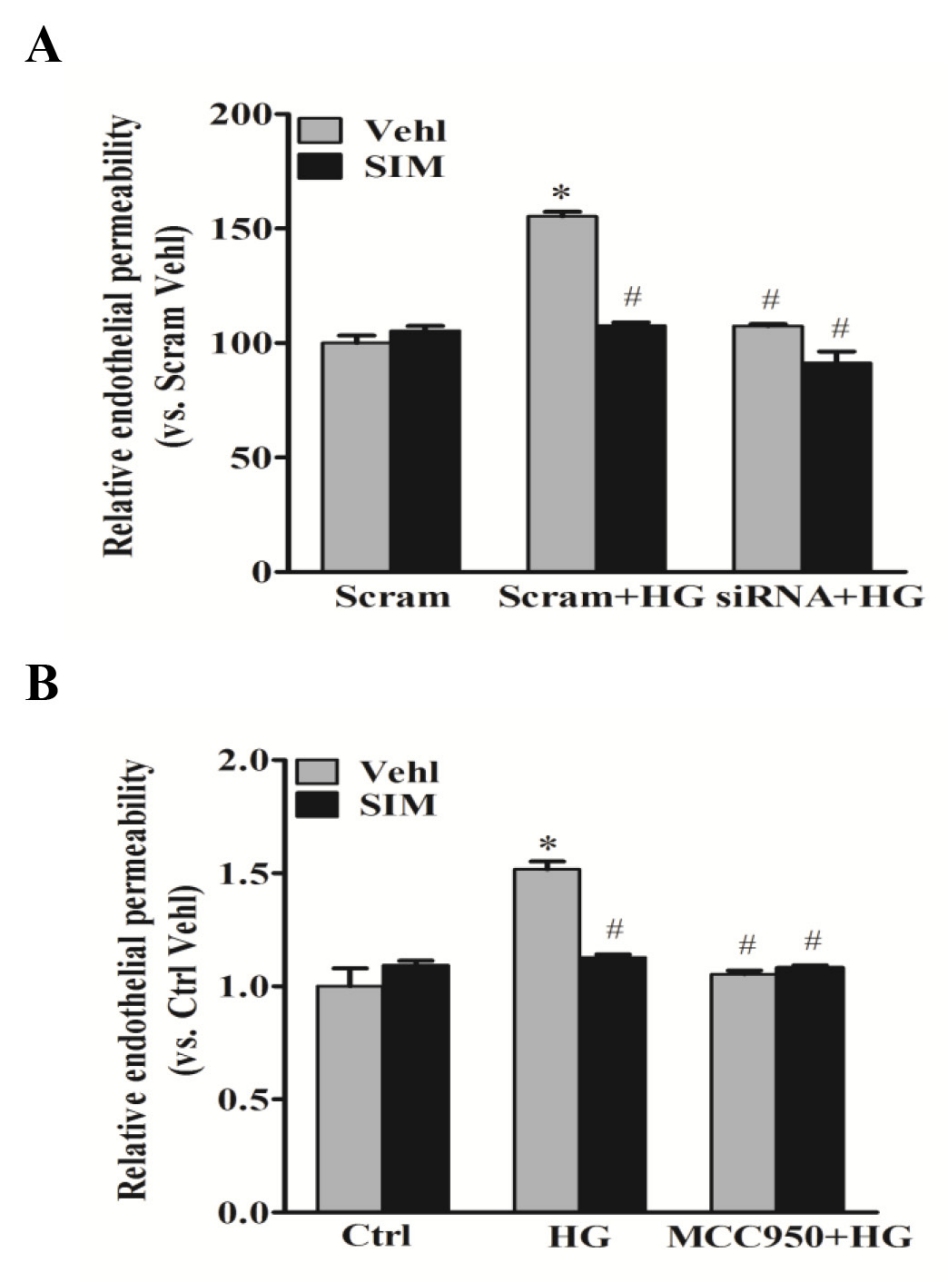
Simvastatin inhibited high glucose-induced endothelial hyperpermeability RAECs were stained by FITC-dextran to test cell permeability. Summarized data showing the role of simvastatin (SIM, 5μM) on the relative permeability of endothelial cell monolayers in the presence or absence of NLRP3 siRNA **(A)** or MCC950 **(B)**. *P<0.05 *vs.* Scram Vehl or Ctrl Vehl; ^#^*P*<0.05 *vs.* HG treated group (n=4).

To confirm the effects of simvastatin on high glucose-induced junction proteins, FITC-dextran was used to test cell permeability through fluorescent microplate reader. As shown in Figure 6A-6B, we observed a significant increase in the relative permeability of endothelial cell monolayers by high glucose, which was effectively prevented in cells pretreated with simvastatin. However, no further effects were found in the relative permeability by the addition with MCC950 or NLRP3 siRNA. Together, these results above suggested that NLRP3 inflammasome mediated the roles of simvastatin on high glucose-induced endothelial hyerpermeability in RAECs.

### Effects of simvastatin on NLRP3 inflammasome-dependent HMGB1

**Figure 7:**
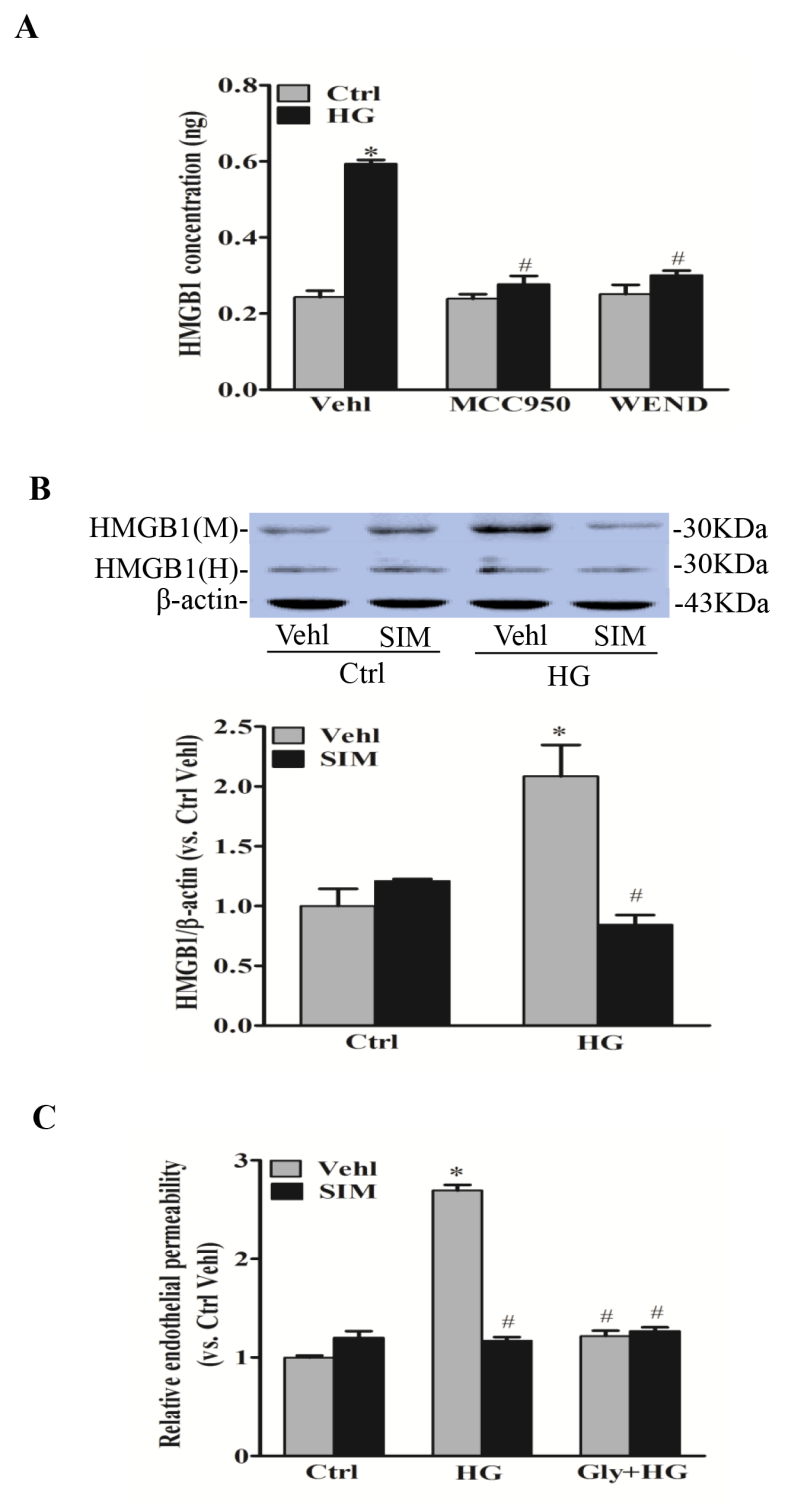
Simvastatin inhibited NLRP3 inflammasome-dependent HMGB1 release **(A)** Summarized data showing the role of NLRP3 inhibitor MCC950 and caspase-1 inhibitor WEND on HMGB1 release from RAECs by commercially available ELISA kit. **(B)** Representative Western blot gel documents and summarized data showing the effect of control or simvastatin (SIM, 5μM) on the expression of HMGB1 or β-actin in either cell culture medium (M) or homogenized cytoplasm (H) of RAECs. **(C)** Summarized data showing the role of simvastatin (SIM) on the relative permeability of endothelial cell monolayers in the presence or absence of the pretreatment of HMGB1 inhibitor glycyrrhizin (130μM). *P<0.05 *vs.* Ctrl Vehl; ^#^*P*<0.05 *vs.* HG treated group (n=4).

HMGB1 is a member of high mobility group nuclear proteins, which is constitutively expressed in the nucleus of eukaryotic cells [20]. Recent studies have demonstrated that HMGB1 is one of the major DAMPs (damage associated molecular patterns), which can be derived from NLRP3 inflammasome activation and increases permeability of the endothelial cell monolayers [21]. By commercially available ELISA kit, we confirmed that HMGB1 release was significantly reduced by NLRP3 inhibitor MCC950 and caspase-1 inhibitor WEND under hyperglycemic condition (Figure 7A). Next, we tested the role of simvastatin on NLRP3 inflammasome-dependent HMGB1 release upon high glucose stimulation. As shown by the increased protein expression of HMGB1 in the culture medium, high glucose treatment increased HMGB1 release from RAECs (Figure 7B). Moreover, high glucose-induced HMGB1 release was dramatically inhibited by simvastatin treatment. We also noticed that HMGB1 activity inhibitor glycyrrhizin prevented high glucose-induced endothelial permeability, and simvastatin showed similar effects on endothelial permeability with simvastatin combined with glycyrrhizin (Figure 7C). Together, this suggested that simvastatin inhibited high glucose-induced endothelial permeability through NLRP3 inflammasome-dependent HMGB1 release.

### Simvastatin prevented vascular hyperpermeability in the myocardium in rats

**Figure 8:**
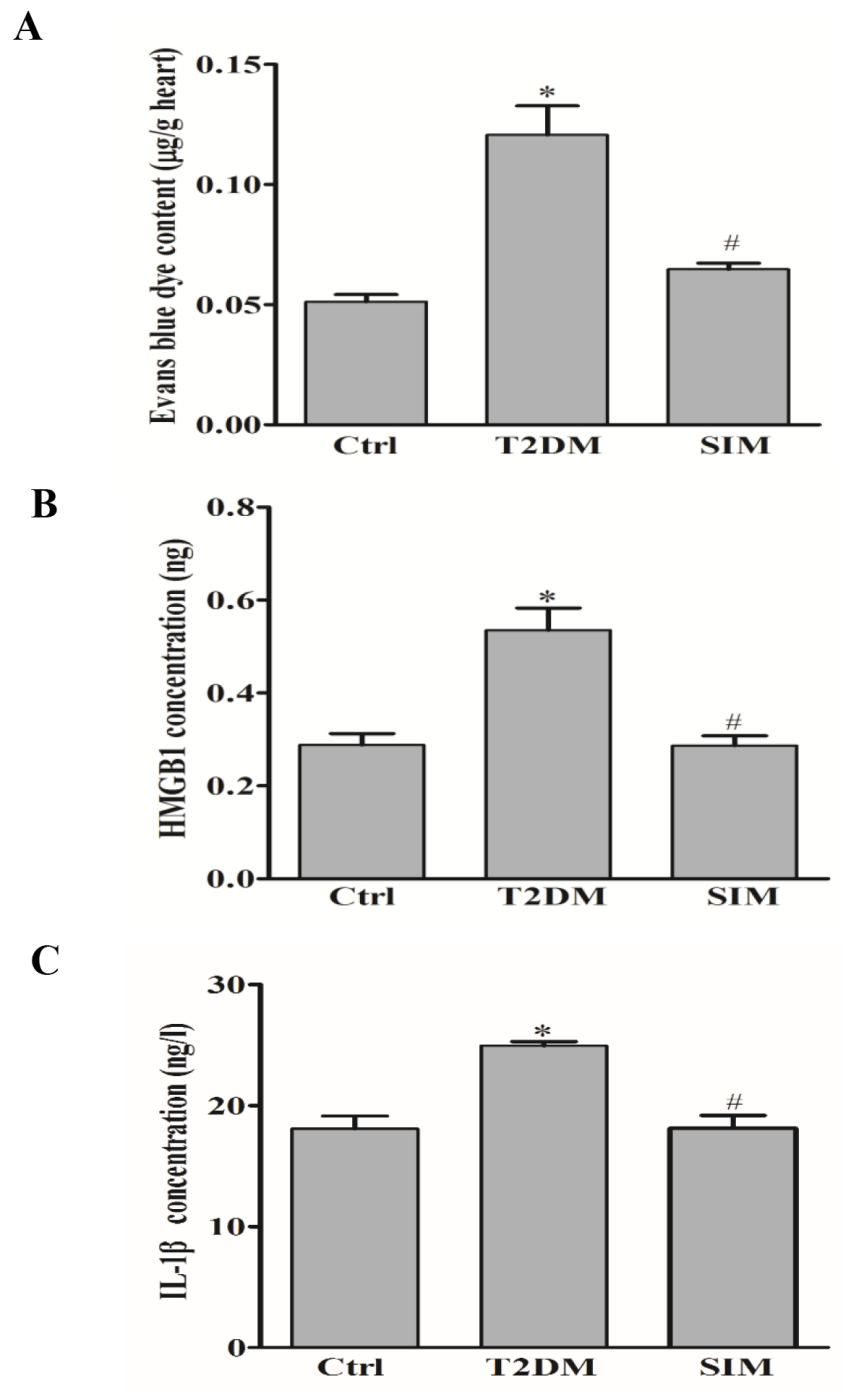
Simvastatin prevented vascular hyperpermeability in rat myocardium Type 2 diabetic rats (T2DM) produced by a high-fat diet and low dose streptozotocin (40mg/kg) were intravenously injected with Evans blue dye. **(A)** Summarized data show that the effect of simvastatin (SIM, 5μM) on the leakage of Evans blue dye from plasma into the interstitial space in rat heart. Summarized data show the level of HMGB1 **(B)** and IL-1β **(C)** in the rat serum by commercially available ELISA kit. *P<0.05 *vs.* Ctrl; ^#^*P*<0.05 *vs.* T2DM rats (n=6-12).

To investigate the protective effect of simvastatin on vascular leakage in rat heart *in vivo*, the rats were intravenously injected with Evans blue dye and leakage of Evans blue dye from plasma into the interstitial space was quantified. As shown in Figure 8A, diabetic rats by a high-fat diet and low dose streptozotocin had significantly higher vascular permeability to intravenously injected Evans blue dye in the heart compared to normal rat. Simvastatin significantly reduced vascular permeability compared to diabetic rats. In addition, diabetic rats had higher release of HMGB1 and IL-1β in the serum compared to normal rat (Figure 8B and 8C). Simvastatin significantly reduced HMGB1 and IL-1β level, which suggested that simvastatin ameliorated vascular pathology in T2DM rats through NLRP3 inflammasome dependent HMGB1 release.

## DISCUSSION

The present study for the first time demonstrated that simvastatin inhibited vascular permeability in RAECs via enhancement of tight junction protein ZO-1 and adherens junction protein VE-Cadherin in endothelial cell monolayers. Notably, the inhibition of HMGB1 release by simvastatin resulted from the inactivation on NLRP3 inflammasome, which was associated with recovery of diabetes-induced vascular permeability. Our data thus suggested that inhibition of NLRP3 inflammasome and consequent improvement of vascular permeability are important determinants of vascular endothelial cell protection by statins.

Endothelial dysfunction is developed at the very early stage of diabetes mellitus with multifactorial mechanism, including altered cell signaling, increased oxidative stress [22], proinflammatory activation [23], and mitochondrial dysfunction [24]. Recent studies indicate that the inflammasome plays important roles in regulating inflammatory cell functions during the development of diabetes [25]. In the present study, our data first demonstrated that high glucose stimulation induced the NLRP3 protein expression and the colocalization of NLRP3 with ASC in RAECs. Biochemical analysis further demonstrated that high glucose increased the caspase-1 activity and IL-1β production in RAECs. Therefore, these results indicate that the NLRP3 inflammasome were functioning in RAECs and that high glucose stimulation can lead to their activation. Our results are consistent with previous findings that NLRP3 inflammasome can be activated by hyperglycemia in cultured mouse vascular endothelial cells or human umbilical vein endothelial cells [17, 26]. In addition, NLRP3 inflammasome are activated in endothelial cells under different pathological conditions such as LPS and ATP, shear stress and adipokine visfatin [27, 28]. Thus, the NLRP3 inflammasome plays a central role in endothelial dysfunction in response to various stimuli and therefore blocking this inflammasome or its key effectors has shown promise for diabetic therapy.

Statins are the most widely prescribed drug class in North America and are used for the prevention and treatment of cardiovascular disease risk [11]. Several recent studies have shown that simvastatin, inhibitor of HMG-CoA reductase, is not only used to treat atherosclerotic disease by blocking cholesterol biosynthesis, but also having an essential effect on anti-inflammation and then preventing endothelial dysfunction [29]. However, the role of simvastatin on NLRP3 inflammasome in endothelial cell is not yet understood. MCC950 was a novel diarylsulfonylurea-based compound that acts as a selective inhibitor of NLRP3 inflammasome activity with no inhibition on the AIM2, NLRC4 or NLRP1 inflammasomes, suggesting that MCC950 specifically blocked NLRP3 inflammatory pathway [30]. The Supplementary Figure 2 demonstrated that the activation of NLRP3 inflammasome pathway induced by high glucose shared similar characteristics with LPS and ATP. Here, we demonstrated for the first time that by using MCC950 and gene silencing for NLRP3 inflammasome, simvastatin inhibited high glucose-induced NLRP3 inflammasome formation and activation. The anti-inflammatory effect of statins may be a key pleiotropic effect that improves cardiovascular disease risk. However, a series of findings have shown that statins could promote insulin resistance by activating the NLRP3 inflammasome, which is associated with an increased risk of new onset diabetes [31]. The effects of statins on adipocytes, myocytes or hepatocytes are speculated to be more relevant to induction of insulin resistance, and the protective effects of statins on endothelial cells that are more relevant to cardiovascular disease. Thus, statin treatment should not be withheld in those at high risk of cardiovascular disease for the relatively minor concern of progression to diabetes. In current study, the anti-inflammatory effects of simvastatin through NLRP3 inflammasome in RAECs provide the evidence regarding the benefits of statin therapy in reducing major cardiovascular events.

Our previous study demonstrated that simvastatin inhibited Rac1 and Rac1-dependent NADPH oxidase activity to exert their beneficial effects in atherosclerosis [12]. Thus, we hypothesize that NADPH oxidase-dependent O_2_^−^ generation mediates the role of simvastatin on NLRP3 inflammasome. The current study found that simvastatin decreased O_2_^−^ formation and inhibited the NLRP3 inflammasome activation via NADPH oxidase-dependent O_2_^−^ production in RAECs. It was consistent with the report that endogenous ROS produced primarily contributes to NLRP3 inflammasome formation and activation [19]. It was also reported that Rac at the cell membrane is a necessary component of the NADPH oxidase complex that drives O_2_^−^ generation, a key component of ROS that are known to be endothelial barrier-disruption [32]. Thus, it is possible that simvastatin plays a protective role in endothelial tight junction through inhibiting NLRP3 inflammasome regulated by NADPH oxidase-dependent O_2_^−^ production.

Endothelial cells are connected to each other by junction proteins including tight junction and adherens junction proteins, such as ZO-1 and VE-Cadherin [33, 34]. The junction proteins are essential to regulating the integrity of the endothelial monolayer and playing a dominant role in the stability of endothelial cell contacts. The disruption of junction proteins occurs in the early-stage of vascular enhanced endothelial permeability [35], which represents one of the initial pathologic processes leading to endothelial dysfunction. In the present study, our data demonstrate that the protein expressions of ZO-1 and VE-Cadherin in RAECs were significantly reduced under high glucose, whereas such decreases were significantly attenuated in simvastatin treatment through NLRP3 inflammasome. Furthermore, we demonstrated that simvastatin recover the permeability of endothelial cell staining by FITC-dextran via NLRP3 inflammasome. Together, our data strongly suggest that recovery of inter-endothelial junction integrity and the consequent endothelial barrier dysfunction in diabetes are associated with endothelial inflammasome inhibition by simvastatin. To our knowledge, these data for the first time link endothelial protection of simvastatin to improvement of endothelial tight junction.

To further clarify the mechanism underlying the simvastatin on endothelial permeability, HMGB1 as a novel permeability factor *in vitro* and *in vivo* was assayed. HMGB1 is a ubiquitous nuclear and cytosolic protein and released by damaged or necrotic cells during sterile inflammation and infection. It has been shown that caspase-dependent HMGB1 release is regulated by NLRP3 inflammasomes, which may represent a very early signaling event during atherogenesis [36]. Our data also confirmed that HMGB1 release was significantly reduced by NLRP3 inhibitor MCC950 and caspase-1 inhibitor WEND under hyperglycemic condition. Additionally, the release of HMGB1 promotes the secretion of IL-1β maturation via NLRP3-inducing caspase-1 pathway [37]. Our data showed that simvastatin inhibited HMGB1 release in endothelial cells stimulated by high glucose. Recent studies have shown that HMGB1 is released from endothelial cells as an autocrine or paracrine to cause junction protein disruption in endothelial cell monolayers [8]. HMGB1 increases the permeability of enterocytic monolayers and impairs intestinal barrier function in mice [38]. Our data further demonstrate that endothelial permeability induced by high glucose was mediated by NLRP3-dependent HMGB1 release, which accounted for the mechanisms about endothelial protective role of simvastatin via NLRP3 inflammasome. In human and mouse endothelial cells, HMGB1 enhances permeability of endothelial monolayers possibly via advanced glycation end products (RAGE)-mediated pathway [39]. Future studies will further detect whether the RAGE-mediated pathway is involved in protection of simvastatin in hyperglycemia-induced endothelial dysfunction.

Vascular endothelium is a semipermeable membrane composed of vascular endothelial cell monolayer, regulating the exchange balance of nutrients and metabolic products between the inner and outer of vascular endothelium [40]. In present study, we further confirm the protective role of simvastatin on vascular leakage in the heart of diabetic rats injected with Evans blue dye. Moreover, simvastatin significantly reduced HMGB1 level in the serum of diabetic rats. Thus, the present study provided direct evidence showing that high glucose treatment enhanced the vascular permeability in the myocardium *in vivo*, which was indeed ameliorated by simvastatin through lowering NLRP3 inflammasome dependent HMGB1. Further study will be done to test whether simvastatin ameliorates vascular pathology in T2DM rats.

In summary, the present study demonstrated that simvastatin inhibited NLRP3 inflammasome in RAECs through inhibiting NADPH oxidase-dependent O_2_^−^ production. This effect of simvastatin is associated with its inhibitory action of HMGB1 release, which results in the recovery of tight junction and vascular permeability. These findings increase our understanding of the mechanisms underlying the anti-inflammatory effects of statins on vascular pathology, which are beyond their lipid-lowering properties.

## MATERIALS AND METHODS

### Cell culture and reagents

The rat aortic endothelial cells (RAECs) line was purchased from American Type Collection Center (ATCC). RAECs were cultured in RPMI medium 1640 basic (1×) (Gibco, USA), containing 10% of fetal bovine serum (Gibco, USA) and 1% penicillin–streptomycin (Gibco, USA). The cells were incubated in 5% CO_2_ at 37°C.

Simvastatin (sigma) was dissolved in 95% ethanol and 0.1 M NaOH, heating at 50°C for 2 h, and neutralizing with HCl to pH 7.2 as described previously [11]. HMGB1 inhibitor glycyrrhizin (Gly) was purchased from Aladdin was dissolved in 1% ethanol and adjusted to PH7.2 with 1M NaOH [8].

### RNA interference of NLRP3

Small interference RNAs (siRNAs) were commercially available (QIAGEN), and the target sequence was as follows: AAGGCAGACCATGTGGATCTA. The sequence for NLRP3 siRNA was confirmed to be effective in silencing NLRP3 gene in different cells by the company. The scrambled small RNA has been also confirmed as non-silencing double stranded RNA and was used as a control in this study. Transfection of NLRP3 siRNA was performed using the GeneTran™ III(BIOMIGA) according to the manufacturer’s instructions, and the efficiency of RNA interference on the expression of NLRP3 protein was confirmed with Western blot analysis.

### Western blot analysis

Western blot analysis was performed as we described previously [41]. Cells were washed twice with phosphate-buffered saline (PBS) and scraped in RIPA lysis buffer (Beyotime). The lysates were centrifuged at 5000×g for 10 minutes at 4°C and the supernatant was collected. Microsomes and cytosols were isolated by a differential centrifugation of the homogenate at 10,000 ×g for 20 min and then at 100,000 ×g for 90 min. The pellet (microsomes) was resuspended in lysis buffer and the supernatant (cytosol) was also collected. The total, cytoplasmic and membrane protein concentration were tested by BCA protein quantitative kit (KeyGEN BioTECH). After boiling for 5 min at 95°C in a 5× loading buffer (BU), the prepared proteins were separated by 6% or 10% sodium dodecyl sulfate-polyacrylamide gel electrophoresis (SDS PAGE) at 80 V for 30 min and then 120V for 1 h, transferred onto polyvinylidene fluoride membranes (PVDF membrane, 0.45 μm, Millipore Co.Ltd.) at 200 mA for 1 h, and then blocked with nonfat milk (5% *w*/*v*) in Tris-buffered saline with Tween 20 for 1 h. The membranes were probed with primary antibody of anti-HMGB1 (1:1000; Proteintech) or anti-ZO-1 (1:500; BOSTER), anti-VE-Cadherin (1:1000; Affinity), anti-β-actin (1:300; BOSTER) overnight at 4°C. After three washed by Tris-buffered saline with Tween 20, the membranes were incubated with Goat Anti-Mouse IgG or Goat Anti-Rabbit IgG (1:3500;BOSTER) for 1.5 hours at room temperature. The immunoreactive bands were detected by chemiluminescent detection systems with LumiGlo and Peroxide (1:1, BU). The density of the bands was analyzed using ImageJ software (NIH, Littleton, CO, USA).

### ELISA

Cells were seeded in 96-well plates at a concentration of 10^4^ cells /well and incubated until 50-60% confluent. After the treatment, cell lysates and cell supernatants were collected and caspase-1activity and IL-1β production was respectively measured by commercially available ELISA kit (AOGENE) according to the protocol described by the manufacturer. Data represent the mean ± standard deviation (SD) from 3 independent experiments.

### Immunocytochemistry

RAECs were grown on glass substrate culture dish for confocal laser scanning (NEST). After rinsed in PBS, cells were fixed with 4% paraformaldehyde (PFA) in PBS for 15 min at room temperature and blocked with 5% BSA for 90min. The cells coverslips were probed with primary antibody of anti-ZO-1 (1:200 BOSTER) overnight at 4°C and incubated for 1 hour with CY3-goat anti-rabbit IgG (1:75 BOSTER) after washing three times with PBS. The slices were visualized through scanning on an Olympus laser scanning confocal microscope (LSM700). In addition, dual-staining was used to detect the colocalization of NLRP3 and ASC, which was analysed by the Image-Pro Plus 6.0 software. The summarized co-localization efficiency data were expressed as Pearson correlation coefficient (PCC) as previous described [10].

### Measurement of O_2_^−^ production

Total O_2_^−^ production in RAECs was measured using the fluorescent probe dihydroethidium (DHE, Sigma, CAS 104821-25-2). The RAECs were cultured in 24-well plates in 5% CO_2_ at 37°C. After treatment, each well was washed twice with 2mL PBS, meanwhile DHE was diluted with serum free-RPMI 1640 medium without phenol red to a final concentration of 10μM. 1mL dilution was evenly added to each well, and incubated in 5% CO_2_ at 37°C without light for 30 min. After the incubation, RAECs were washed 3 times with 1mL PBS, red fluorescence images were captured by fluorescence microscopy (Olympus IX53). The intensity of fluorescence was analyzed and processed by ImageJ software and the ratio of fluorescence intensity to that at basal level was quantified to show O_2_^−^ level.

### Flowcytometry assay

The expression of ZO-1 on the cell membrane of intercellular tight junctions was also assessed by flowcytometry. RAECs were cultured in 24-well culture plates and treated as indicated. RAECs were washed with PBS and then blocked with 1% bovine serum albumin (BSA) for 10 min at 4°C. After two washes, RAECs were incubated with rabbit anti-ZO-1 (1:200 BOSTER) for 1 hour, followed by incubation with FITC labeled goat anti-rabbit IgG (1:75 BOSTER) for 30 min. After another three times 5-min washes in PBS, the cells were trypsinized by 100 μl 2×trypsine for 1 min and terminated by 500μl RPMI 1640 media. Then the cells in each well were suspended and run on a flow cytometer (BD Accuri) and analyzed with FlowJo 7.6 software.

### Vascular endothelial permeability

Cells were seeded on polyester 0.4μm pore membranes in Transwell inserts (Millipore) and placed in 24-well plates, and incubated for 24 hours to allow the growth of a confluent monolayer. Monolayers were treated as indicated for 24h, and then the transwell inserts were moved into new wells with 500μl fresh medium (RPMI 1640 medium, no phenol red; Solarbio Beijing). Monolayers were incubated at 37°C for 2 hours after added 10μl 10 mg/ml 10-kDa fluorescein isothiocyanate (FITC)-Dextran (Sigma) to the upper chamber. Specifically, aliquots of the lower and upper chambers were withdrawn, added to a 96 well plate, and then measured for fluorescence intensity at excitation/emission of 485/530nm in a fluorescent microplate reader (FL×800, BIO-TEK Instruments; POLAR star Omega). Permeability was calculated as Pa=[A]/t×1/A×V/[L],where [A] is the fluorescence intensity of the lower chamber, A is the area of the transwell (cm^2^),V is the volume of the lower chamber, and [L] is the fluorescence intensity of the upper chamber. Data represent the mean standard deviation (SD) from 3 independent experiments.

### Vascular permeability assay

Male Sprague-Dawley rats weighing 120-150g were purchased from the Laboratory Animal Center of Nanjing Qinglongshan. Animal handling and experimental procedures were approved by the ethic committee of China Pharmaceutical University, in accordance with the Guidelines of Animal Experiment set by the Bureau of Sciences and Techniques of Jiangsu Province, China [NO.SYXK2007-0025].

The rats were randomly allocated into 2 groups: a normal diet group (ND, n=12), and a type 2 diabetes mellitus group (T2DM, n=12). The rats were fed either a normal diet or a high fat diet for 13 weeks. After 4 weeks of dietary manipulation, the rats in T2DM group were received intraperitoneal injection of streptozocin (STZ, Sigma, USA) at a dose of 40mg/kg (dissolved in 0.1M sodium citrate buffer, pH4.5). After 2 weeks of fasting blood glucose monitoring at one week intervals, a diabetic rat model was confirmed when hyperglycemia was greater than 11.1 mmol/L. Subsequently the rats in T2DM group were randomly divided into two groups: (1) STZ injected alone; (2) STZ injected and intervention with simvastatin (20mg/Kg, qd) by intragastric administration for two weeks. All rats were maintained in a standard environment at a controlled temperature (25±2°C).

Assessment of vascular permeability in rat hearts was performed as described previously [42]. Rats were injected with 60mg/kg of Evans blue solution via the tail vein, and anesthetized by 3% chloral hydrate after 2 hours, subsequently, injected with PBS to remove the residual blood and Evans blue solution in heart. The dissected heart were dipped in Normal saline-acetone (V_NS_:V_acetone_=3:7) to grind into 10% homogenate (M_heart_/V_NS-acetone_), overnight at 4°C, and the supernatant was collected after centrifuging at 1250×g for 15 minutes. The amount of Evans blue in heart was quantitated by UV-specrophotometer at 620 nm.

### Statistics

Data are presented as means ± SE. Significant differences between and within multiple groups were examined using ANOVA for repeated measures, followed by Duncan’s multiple-range test. The Students *t* test was used to detect significant differences between two groups. *P*<0.05 was considered statistically significant.

## SUPPLEMENTARY MATERIALS FIGURES


