# Supplementary material for: Protection by simvastatin on hyperglycemia-induced endothelial dysfunction through inhibiting NLRP3 inflammasomes
Source: Oncotarget. 2017 Aug 24;8(53):91291–305. doi: 10.18632/oncotarget.20443 (PMC5710924; doi:10.18632/oncotarget.20443)
Supplement: Supplementary file 1 [file oncotarget-08-91291-s001.pdf]

Protection by simvastatin on hyperglycemia-induced endothelial dysfunction through inhibiting NLRP3 inflammasomes

SUPPLEMENTARY MATERIALS

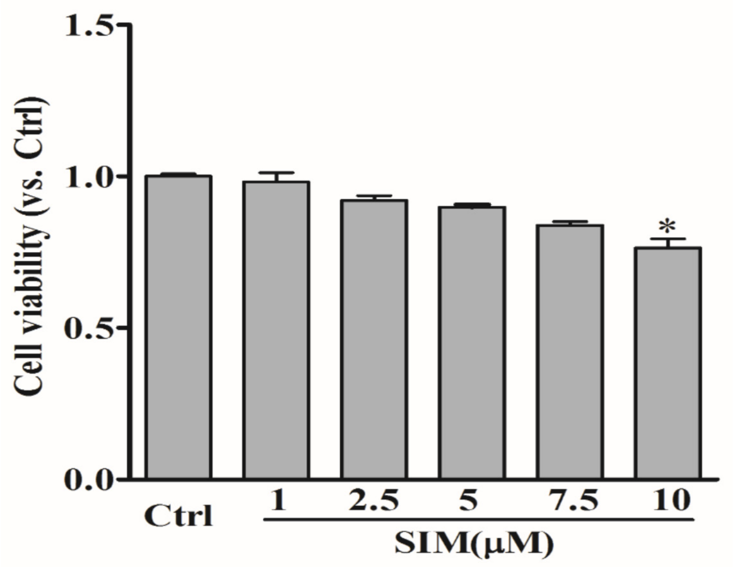

Supplementary Figure 1: Effects of simvastatin (SIM) on cell viability in RAECs. MTT assay showed that SIM dose-dependently inhibited cell viability from 1 uM to 10 uM, but no toxic effects on RAECs at 5 uM. *$P<0.05$ *vs.* Control (Ctrl) (n=3).

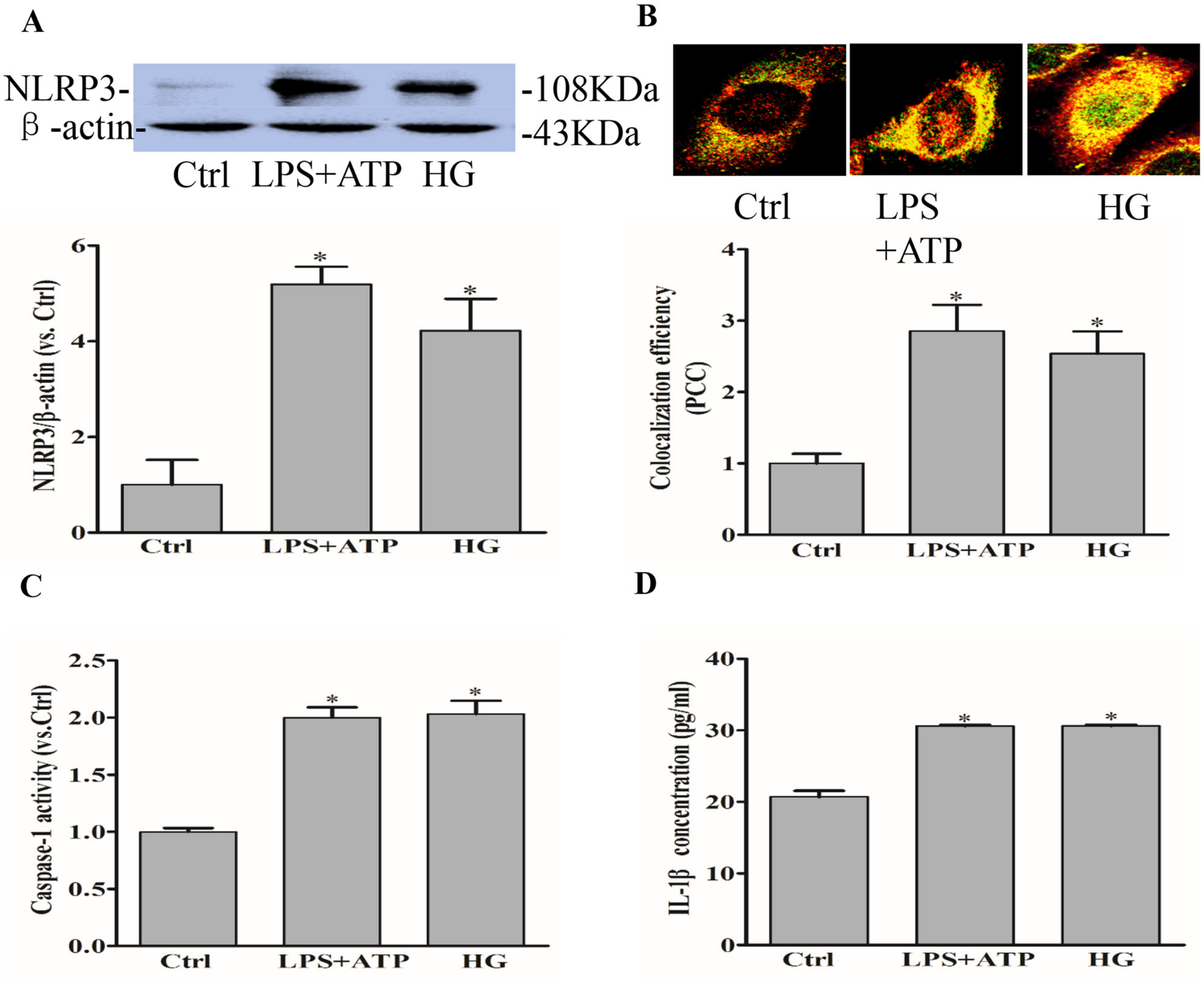

Supplementary Figure 2: Effects of LPS plus ATP and HG on the activation of NLRP3 inflammasomeon. **(A)** Representative Western blot gel documents and summarized data showing LPS plus ATP and HG on NLRP3 expression. **(B)** Representative confocal fluorescence images and colocalization efficiency showing the showing the colocalization of NLRP3 with ASC. **(C)** Summarized data showing caspase-1 activity in RAECs. **(D)** Summarized data showing IL-1β production in RAECs. $^{*}P<0.05$ *vs.* Control (Ctrl) (n=3).
